# Supplementary material for: A Novel Strategy for the Synthesis of High Stability of Luminescent Zero Dimensional–Two Dimensional CsPbBr3 Quantum Dot/1,4-bis(4-methylstyryl)benzene Nanoplate Heterostructures at an Atmospheric Condition
Source: Nanomaterials (Basel). 2023 Oct 7;13(19):2723. doi: 10.3390/nano13192723 (PMC10574592; doi:10.3390/nano13192723)
Supplement: Supplementary file 1 [file nanomaterials-13-02723-s001.zip › nanomaterials-2597135-supplementary.pdf]

nanomaterials

MDPI

Supplementary Materials

A Novel Strategy for the Synthesis of High Stability of Luminescent 0D-2D $CsPbBr_3$ QD/*p*-MSB Nanoplate Heterostructures at an Atmospheric Condition

Yanran Wang [1,2], Ming-Yu Li[1,2,*], Shijie Liu[2], Yuan Ma[2], Bo Sun[2], Liangyu Wang[2], Haifei Lu[2], Xiaoyan Wen[2], Sisi Liu[2,*], Xumin Ding[3]

[1] Donghai Laboratory, Zhoushan Zhejiang 316021, China
[2] School of Science, Wuhan University of Technology, Wuhan, Hubei 430070, China
[3] Advanced Microscopy and Instrumentation Research Center, School of Instrumentation Science and Engineering, Harbin Institute of Technology
* Correspondence: mingyuli.oliver@gmail.com (M.-Y.L.); liusisi0109@hotmail.com (S.L.)

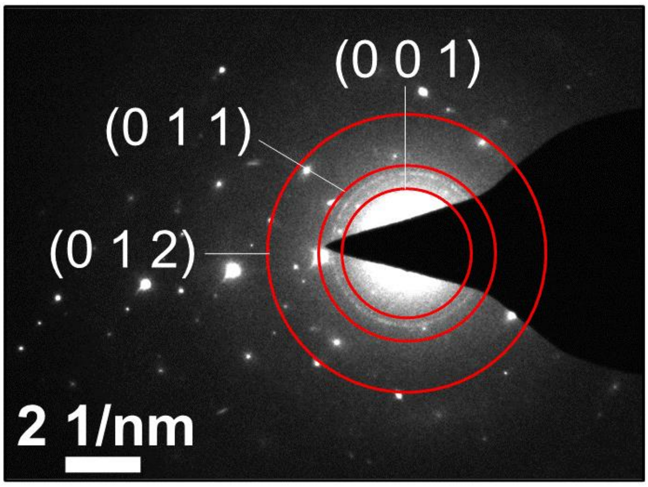

Figure S1. The selected area electron diffraction (SAED) pattern image of the $CsPbBr_3$ QD/*p*-MSB NP solutions by blending with 1 mg/mL *p*-MSB.

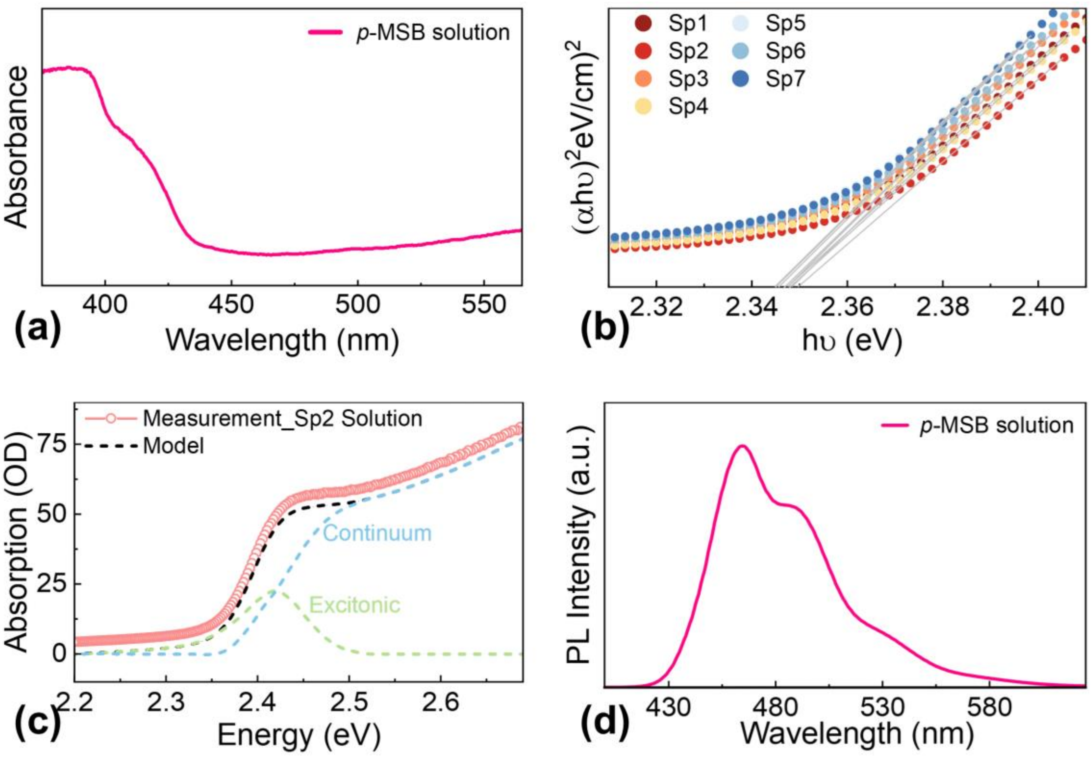

Figure S2. (a) Absorption spectra of *p*-MSB NP solutions. (b) The Tauc plots of $CsPbBr_3$ QD/*p*-MSB NP solutions. (c) Absorption spectrum and Elliott's model of the $CsPbBr_3$ QD/*p*-MSB NP solutions by blending with 1 mg/mL *p*-MSB. (d) normalized PL spectra of *p*-MSB NP solutions.

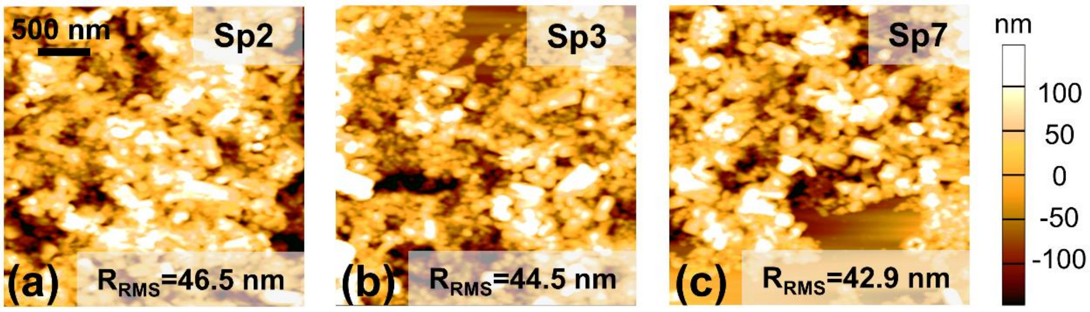

Figure S3. Atomic force microscopy (AFM) images of the samples: (a) Sp2, (b) Sp3, (c) Sp7.

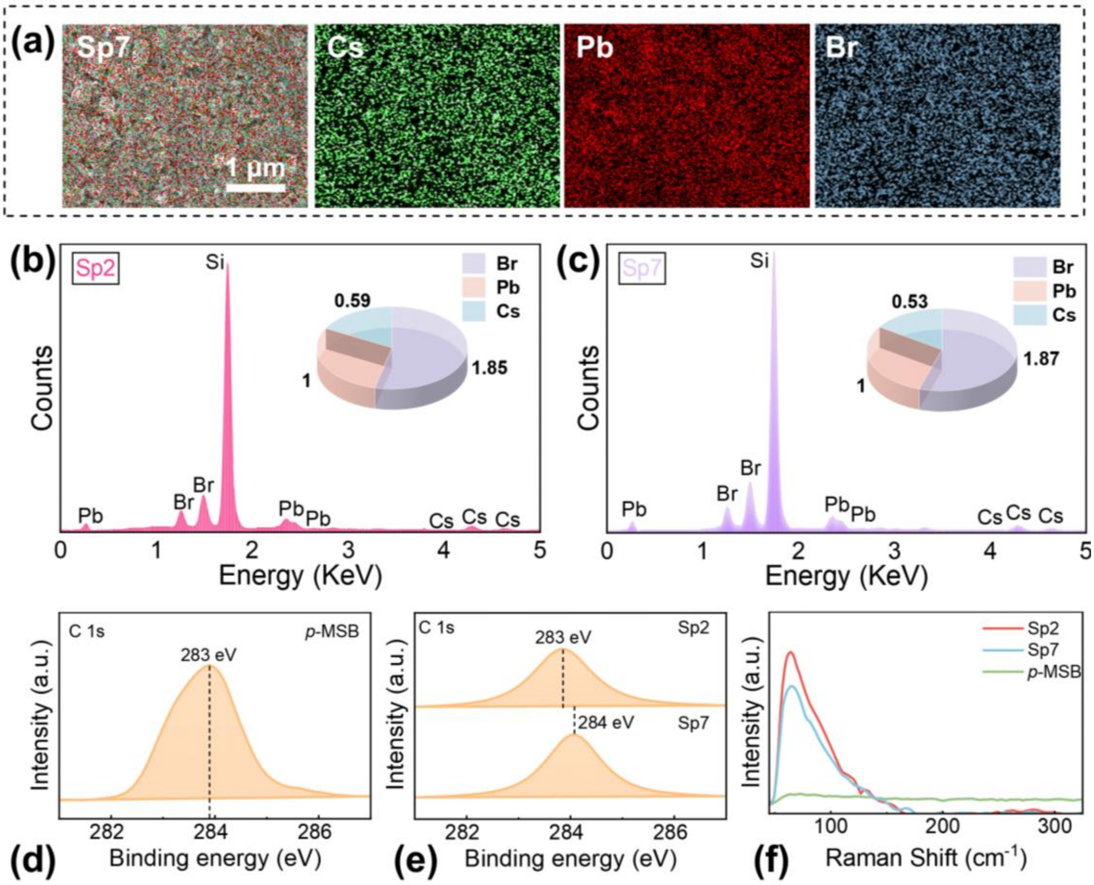

Figure S4. (a) Energy dispersive X-ray spectroscopy (EDS) element maps of the sample Sp7. EDS spectra of the samples (b) Sp2 and (c) Sp7. X-ray photoelectron spectroscopy (XPS) spectra of C 1s signatures on the (d) *p*-MSB and (e) Sp2 and Sp7 samples. (f) Raman spectra of Sp2, Sp7 and *p*-MSB measured with a 532 nm excitation laser.

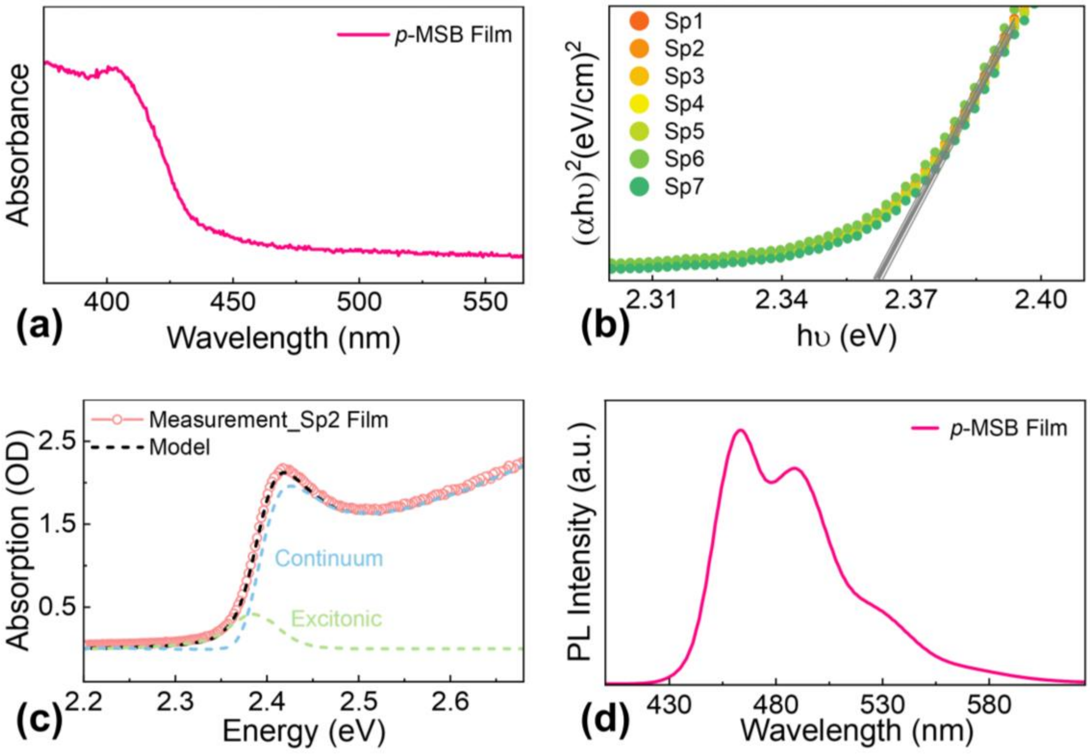

Figure S5. (a) Absorption spectra of *p*-MSB thin films. (b) The Tauc plots of $CsPbBr_3$ QD/*p*-MSB NP thin films. (c) Absorption spectrum and Elliott's model of the $CsPbBr_3$ QD/*p*-MSB NP films by blending with 1 mg/mL *p*-MSB. (d) Normalized PL spectra of *p*-MSB thin films.

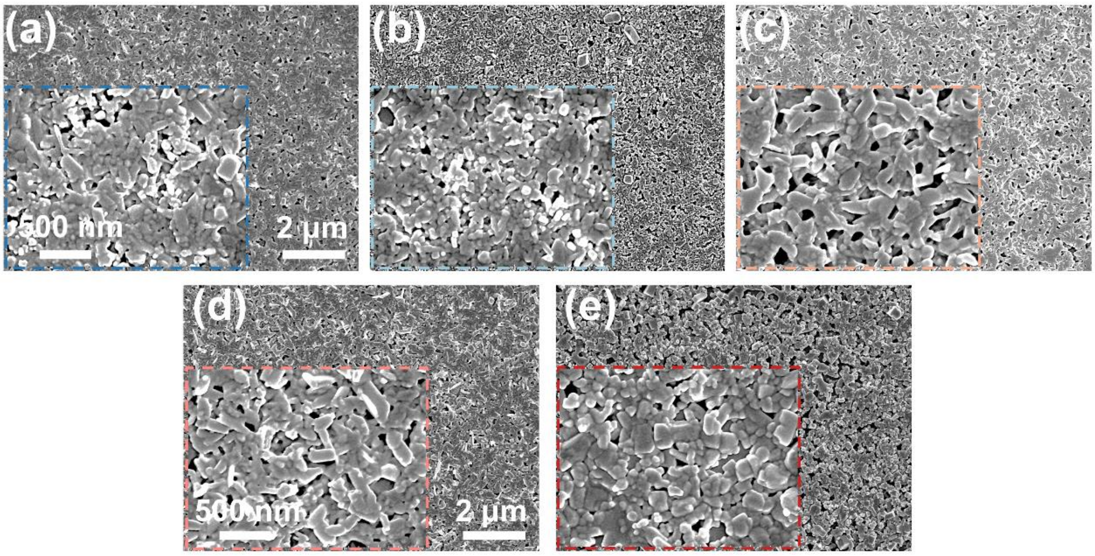

Figure S6. Scanning electron microscope (SEM) images of the hybrid $CsPbBr_3$ QD/*p*-MSB NP thin films with 1 mg/mL *p*-MSB at different annealing temperatures: (a) 25 °C, (b) 40 °C, (c) 60 °C, (d) 80 °C, and (e) 100 °C.

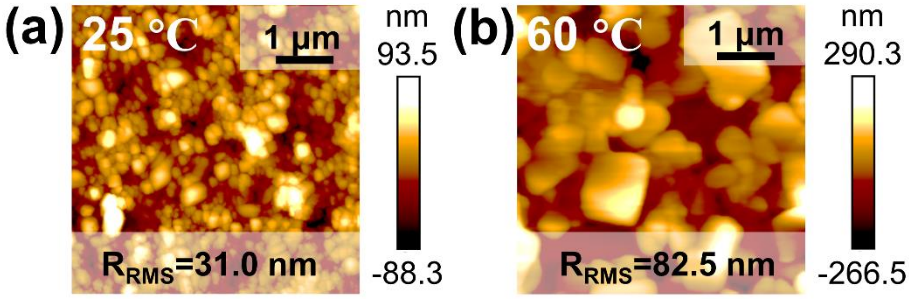

Figure S7. Atomic force microscopy (AFM) of the hybrid $CsPbBr_3$ QD/*p*-MSB NP thin films with 1 mg/mL *p*-MSB at different annealing temperatures: (a) 25 °C and (b) 60 °C.

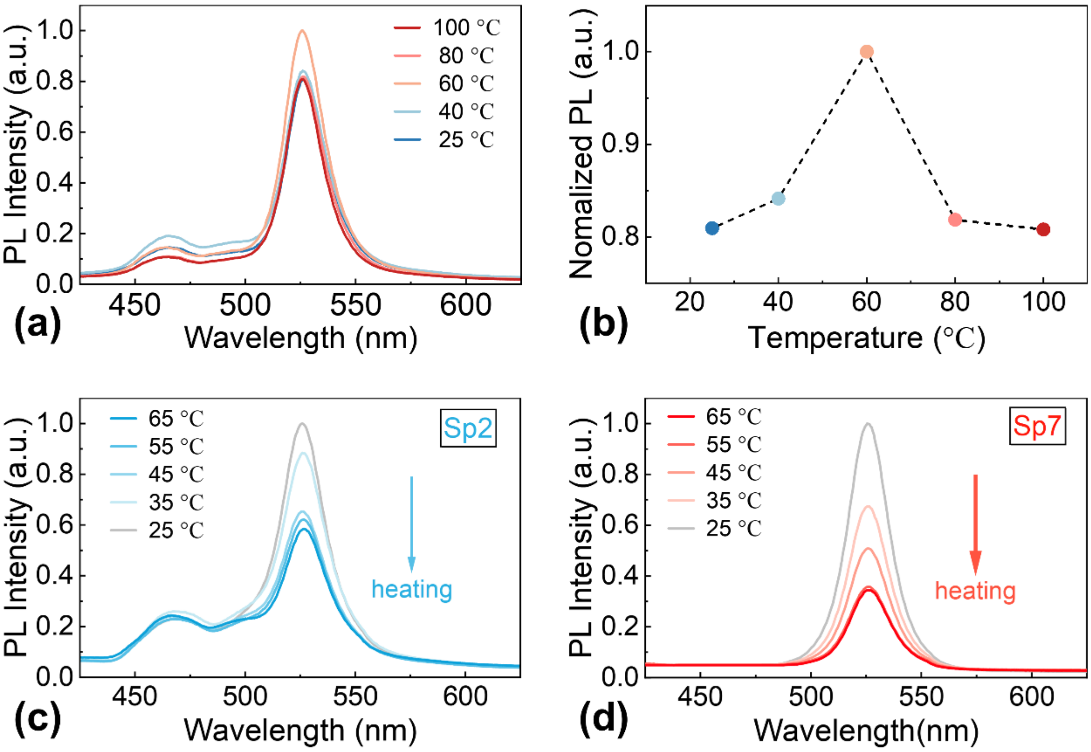

Figure S8. (a) Normalized room-temperature PL spectra and (b) the plots of PL intensities of hybrid $CsPbBr_3$ QD/*p*-MSB NP thin films with 1 mg/mL *p*-MSB fabricated at different annealing temperatures. Normalized PL spectra of the samples (c) Sp2 and (d) Sp7 after heating at various temperatures.

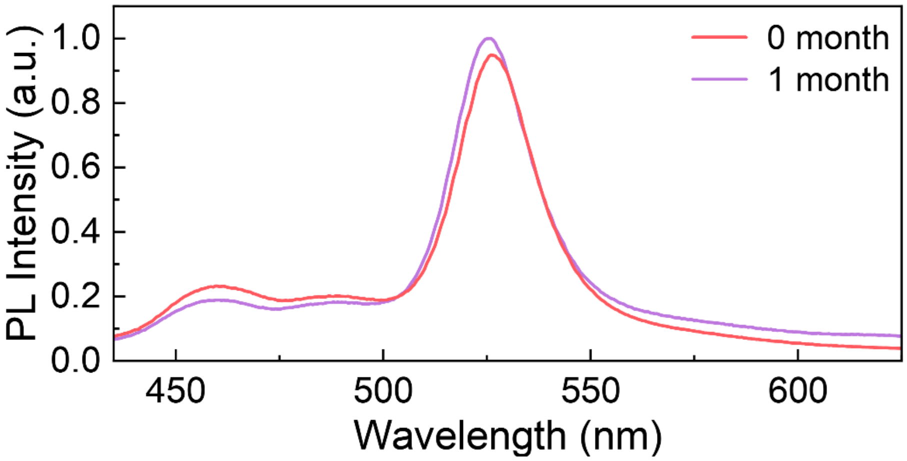

Figure S9. Normalized photoluminescence (PL) spectra of the sample Sp2 as-fabricated (red) and after storing for 1 month (purple) in atmospheric ambient air.

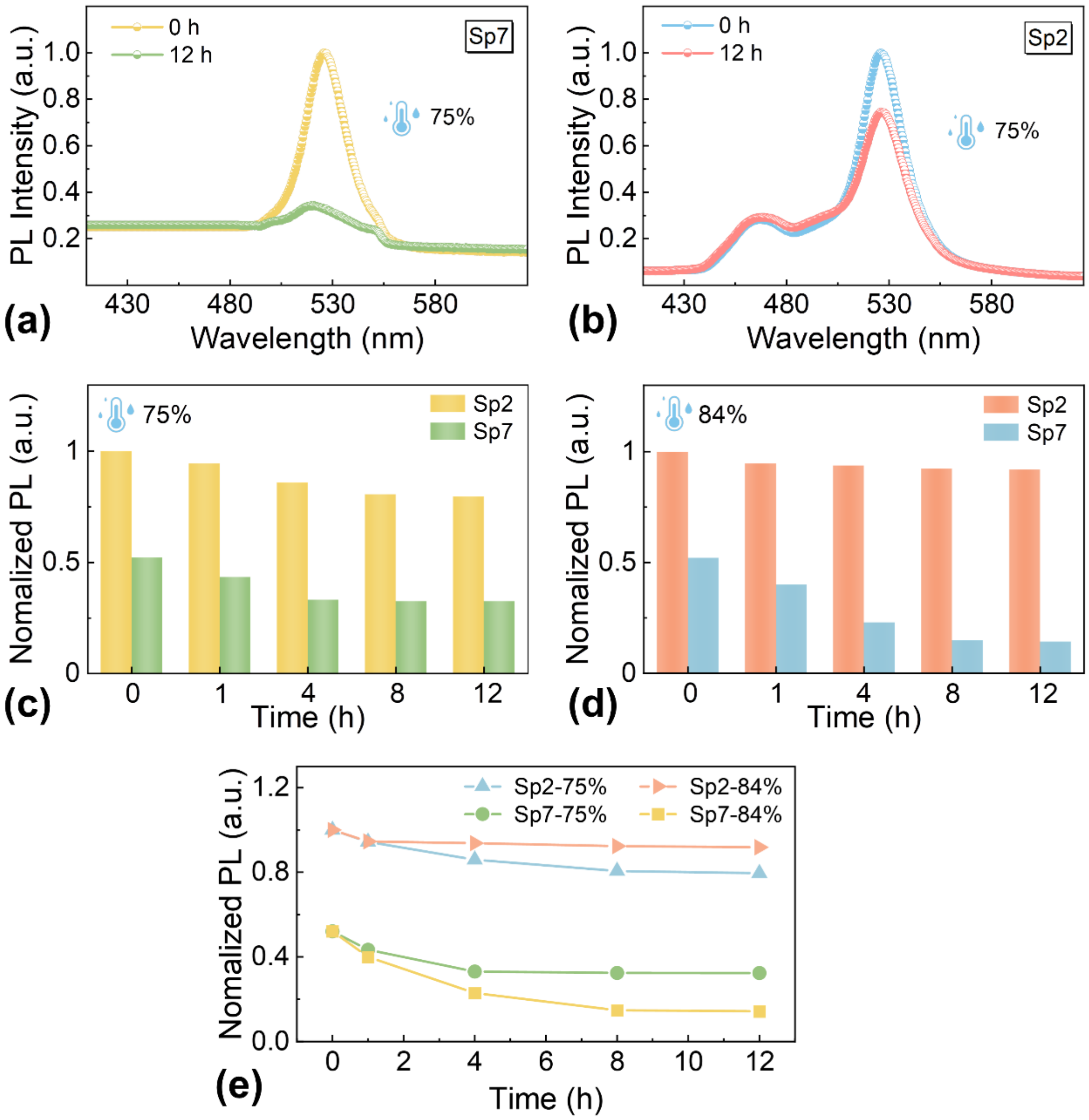

Figure S10. Normalized PL spectra of the sample (a) Sp7 and (b) Sp2 as-fabricated and after storing for 12 h at 75% RH. Normalized PL spectra of the sample Sp7 and Sp2 at (c) 75% RH and (d) 84% RH. (e) The time-dependent PL intensities as a function of relative humidity of the samples Sp2 and Sp7.

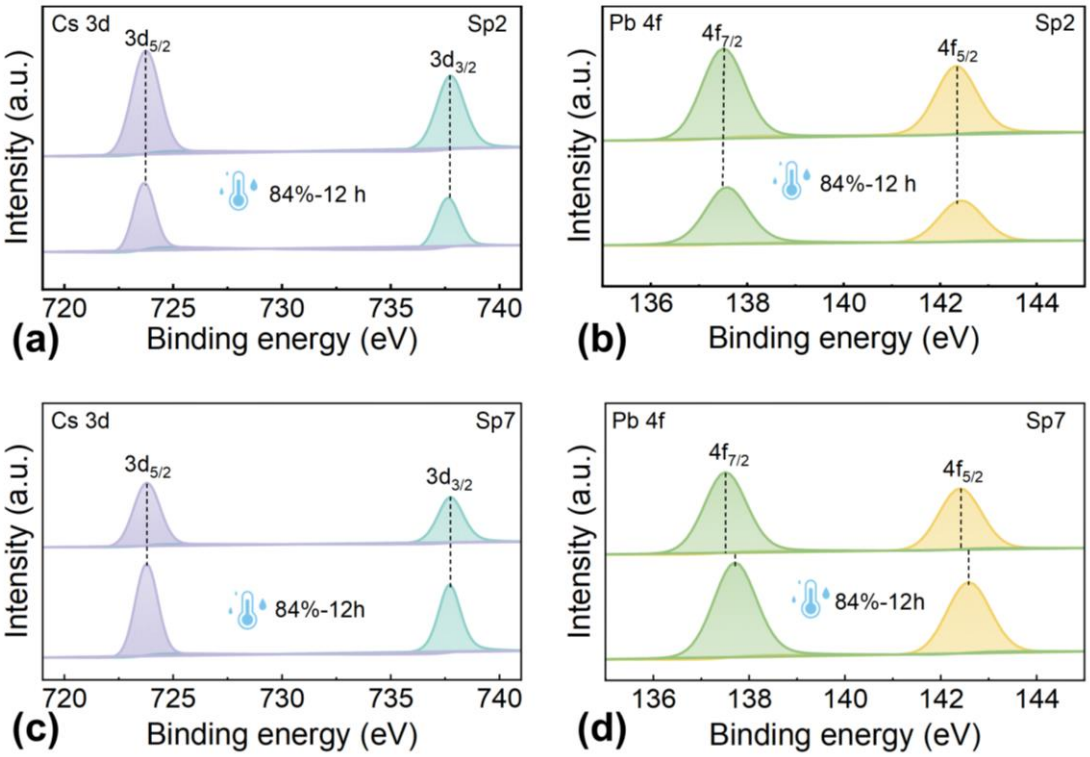

Figure S11. X-ray photoelectron spectroscopy (XPS) spectra of (a) Cs 3d and (b) Pb 4f signatures on the samples Sp2 as-fabricated and after storing for 12 h at 84% RH. (f) X-ray photoelectron spectroscopy (XPS) spectra of (c) Cs 3d and (d) Pb 4f signatures on the samples Sp7 as-fabricated and after storing for 12 h at 84% RH.

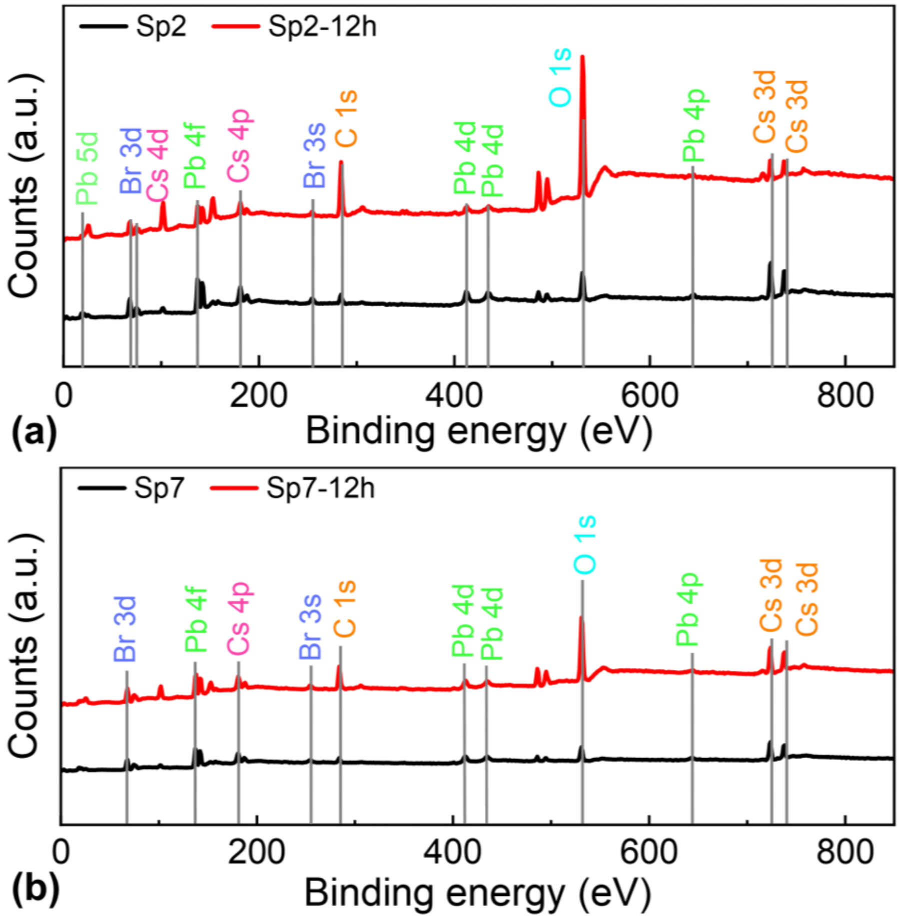

Figure S12. The full-range survey scan XPS spectra of the samples (a) Sp2 and (b) Sp7 as-fabricated and after storing for 12 h at 84% RH.

Table S1. PL Decay Time Constants (τ_1, τ_2, and τ_3) and Average Lifetimes (τ_{avg}) of Sp2 and Sp7.

	A_1(%)	τ_1 (ns)	A_2(%)	τ_2 (ns)	A_3(%)	τ_3(ns)	τ_{avg}(ns)
Sp2	51.84	3.35	40.07	13.44	8.09	53.20	11.43
Sp7	24.25	47.61	56.49	38.28	19.25	5.74	34.27

Table S2. PL Decay Time Constants (τ_1, τ_2, and τ_3) and Average Lifetimes (τ_{avg}) of Sp2 at different annealing temperatures.

	A_1(%)	τ_1 (ns)	A_2(%)	τ_2 (ns)	A_3(%)	τ_3(ns)	τ_{avg}(ns)
25 ℃	45.08	3.18	41.19	13.49	13.73	52.16	14.15
60 ℃	36.83	3.35	44.96	16.09	18.21	55.69	18.97
